# What is it about a cancer diagnosis that would worry people? A population-based survey of adults in England

**DOI:** 10.1186/s12885-017-3963-4

**Published:** 2018-01-24

**Authors:** Philippa J. Murphy, Laura A. V. Marlow, Jo Waller, Charlotte Vrinten

**Affiliations:** Department of Behavioural Science and Health, UCL, Gower Street, London, WC1E 6BT UK

**Keywords:** Cancer, Oncology, Worry, Fear, Screening, Early detection, Prevention

## Abstract

**Background:**

Surveys indicate quite high prevalence of cancer worry in the general population, but little is known about what it is about cancer that worries people. A better understanding of the origins of cancer worry may help elucidate previously found inconsistencies in its behavioural effect on cancer prevention, screening uptake, and help-seeking for symptoms. In this study, we explore the prevalence and population distribution of general cancer worry and worries about specific aspects of cancer previously identified.

**Methods:**

A population-based survey of 2048 English adults (18–70 years, April–May 2016), using face-to-face interviews to assess demographic characteristics, general cancer worry and twelve sources of cancer worry (adapted from an existing scale), including the emotional, physical, and social consequences of a diagnosis.

**Results:**

In general, a third of respondents (37%) never worried about cancer, 57% worried occasionally/sometimes, and 6% often/very often. In terms of specific worries, two thirds would be ‘quite a bit’ or ‘extremely’ worried about the *threat to life* and *emotional upset* a diagnosis would cause. Half would worry about *surgery*, *radiotherapy*, *chemotherapy*, and *loss of control over life*. Worries about the social consequences were less commonly anticipated: just under half would worry about *financial problems* or their *social roles*, and a quarter would be worried about effects on *identity*, *important relationships*, *gender role*, and *sexuality*. Women and younger people reported more frequent worry about getting cancer, and would be more worried about the emotional, physical, and social consequences of a cancer diagnosis (*p* < .001). Those from ethnic minority backgrounds reported less frequent worry about getting cancer than their white counterparts, but would be equally worried about the emotional and physical impact of a cancer diagnosis, and worried more about the social consequences of a cancer diagnosis (*p* < .05).

**Conclusions:**

The majority of English adults worry at least occasionally about getting cancer, and would be most worried about the emotional and physical impact of a cancer diagnosis. Distinguishing between the various worries that cancer can evoke may help inform efforts to allay undue worries in those who are deterred by them from engaging with cancer prevention and early detection.

**Electronic supplementary material:**

The online version of this article (10.1186/s12885-017-3963-4) contains supplementary material, which is available to authorized users.

## Background

Population surveys in the UK and US show that 25–50% of people worry about getting cancer), with 5 to 10% being extremely worried [1–3]. Cancer worry, defined as a negative emotional reaction to the threat of cancer [4], is often operationalised as a unidimensional construct. Studies usually measure its *frequency* [5–7], *intensity* [3, 5, 8], the *physical experiences* associated with this negative emotional reaction, such as fear or anxiety [3, 9], or a combination [5]. However, little is known about whether there could be different aspects of cancer that evoke different worries in people in the general population.

Cancer worry in the general population has been shown to affect uptake of cancer screening [4, 10], help-seeking for possible cancer symptoms [11, 12], and adoption of cancer-preventive health behaviours [1]. However, some of these studies have found that worry is a motivator and others that it is a deterrent. Some authors have suggested that it may be necessary to identify the specific origins of cancer-related worry to make sense of the inconsistencies observed in cancer worry’s behavioural effects [4, 13].

The idea that the behavioural effect of worry is dependent on its source, is consistent with health behaviour theories that assign a central role to efficacy beliefs in dealing with a threat, such as Witte’s Extended Parallel Process Model (EPPM) [14] or Rogers’ Protection Motivation Theory [15]. For example, according to Witte’s EPPM, the response to a perceived threat is dependent on the outcome of an efficacy appraisal, including beliefs about self- and response-efficacy. If efficacy is high, the behavioural response will aim to avert the danger associated with the threat. If efficacy is low, the behavioural response will aim to decrease the fear associated with the threat, rather than the threat itself. Since efficacy beliefs are guided by the nature of the threat, it follows that the behavioural response may differ. An example of this is a US-based study of prostate cancer screening which found that *worry about prostate cancer* was associated with more frequent screening to obtain reassurance, while *fear of screening* led to less frequent screening [16]. This study suggests that deterring effects of screening fears could be addressed by increasing efficacy beliefs about screening (e.g. self-efficacy). If, on the other hand, interventions were developed to reduce worry about prostate cancer, for example, by developing strategies to cope with prostate cancer worry, then this could be expected to result in lower screening uptake. Although this study measured fear of the screening procedure itself rather than fear of screening outcomes, it highlights the need for a more precise and comprehensive definition and operationalisation of ‘cancer worry’ in the general population. Because 1 in 2 people in the UK born after 1960 are expected to develop cancer within their lifetime [17], it is important to understand how cancer-related worries in those without a cancer diagnosis in the general population may affect behaviours such as screening uptake and help-seeking for symptoms to inform efforts to increase informed participation in cancer prevention and early detection. However, as the above study shows [16], interventions to increase efficacy in dealing with the threat of cancer would likely depend on the nature of the worry cancer evokes.

In contrast to population-based studies about cancer screening and public health, studies in cancer patients already tend to operationalise fear of cancer recurrence^1^ as a *multidimensional* construct, including fears about further treatment, loss of autonomy, and the effects of a recurrence on social activities, work, the partner, or the family [18–20]. Studies in those seeking help for possible cancer symptoms also suggest multidimensionality, including fears about the physical and financial consequences of cancer treatment, suffering, body disfigurement, leaving others behind, and death [11, 12, 21, 22]. Thus, in these subpopulations, cancer appears to evoke a wide range of fears.

Evidence that cancer worry may also be multidimensional in the general population comes from a recent systematic review of 102 qualitative studies carried out in the context of cancer screening [23]. In those eligible for screening, cancer worry manifested itself as a general worry about cancer, or as specific worries, such as about the emotional and physical consequences of a diagnosis (for example, chemotherapy and surgery), the social consequences of a diagnosis, or dying from cancer. Thus, this review suggests that cancer worry in the general population may have various specific origins. However, the qualitative nature of this review prevents any conclusions about the prevalence, population distribution, or behavioural effects of these different types of worry.

The present study aims to provide a starting point to exploring the usefulness of distinguishing between different origins of cancer worry in the general population who have not been diagnosed with cancer, by examining their prevalence and whether responses vary by demographic group. We chose to focus on twelve specific cancer worries that were commonly reported in the aforementioned qualitative review of the literature about cancer screening in the general population, and may therefore be hypothesised to be main cancer worries among the general population [23]. We also compare anticipated worries about these twelve aspects of a cancer diagnosis with a commonly-used measure of general cancer worry [24]. Previous studies have shown that general cancer worry is higher in women, in those who are younger, and in those from more deprived or ethnic minority backgrounds [16, 25–27].

## Methods

### Design and procedure

Data were collected as part of the third wave of the Attitudes, Behaviour, and Cancer UK Survey (ABACUS), using home-based face-to-face computer-assisted interviews conducted by market research agency TNS Research International as part of their weekly omnibus survey. This survey uses random location sampling of sample points defined by the 2011 Census small area statistics and the Postcode Address File. At each location, quotas are set for age, gender, working status, and children in the household.

### Participants

Interviews with 2048 adults aged 18–70 years were conducted in the respondents’ homes in England in April and May 2016. This age range was chosen to reflect the adult population in England, but since the survey was part of a module about attitudes towards cancer and cancer screening, the upper age limit was set at 70 years because this is the age at which people stop being invited to cancer screening in England. Ethical approval was obtained from the UCL Research Ethics Committee (registration number 5771/002), and participants consented to participate at the start of the interview. Those who were diagnosed with cancer were excluded from the cancer worry questions to avoid distress.

### Measures

General cancer worry was assessed with the question: ‘How often do you worry about your chance of getting cancer yourself?’, which was scored on a 5-point scale (never to very often). This item was adapted from the US Health and Information National Trends Survey [24] and has been used in many previous studies (e.g. [7, 28, 29]).

The sources of cancer worries described by those eligible for screening in the review [23] showed great overlap with those mentioned by cancer survivors. Thus, we decided to assess these specific worries about cancer using items adapted from the Concerns About Recurrence Scale (CARS) [20]. The CARS is a validated and internally reliable 30-item scale to assess breast cancer survivors’ concerns about recurrence, which we adapted through a process of cognitive interviewing and online piloting with asymptomatic adults in England for use in a general population sample without a previous diagnosis of cancer (see Additional file 1). We included twelve items in our survey that covered a wide range of specific cancer worries that are also reported by those seeking help for possible cancer symptoms [11, 21, 22] or those eligible for cancer screening [23]. Because cancer worry in the general population is predicated on the threat of cancer (for example, evoked by an invitation to cancer screening or the discovery of a symptom that may indicate cancer), all items started with the phrase “If you were diagnosed with cancer, would you worry that…?” and were measured on a 4-point scale (not at all to extremely). An exploratory factor analysis showed items loaded onto two factors: worries about the emotional and physical consequences of a cancer diagnosis (such as worries about the emotional upset that a cancer diagnosis would cause, and about chemotherapy and surgery), and worries about its social consequences (such as worries about the effect on relationships with family and friends, interference with sense of sexuality, and financial problems). The twelve items and factor loadings are presented in Additional file 2. Both subscales had good internal reliability (Cronbach’s α = .88 and α = .85, respectively). We calculated mean sub-scale scores for those with complete data on all twelve items.

Age, gender, ethnicity, marital status, and socioeconomic status were assessed with simple questions. Ethnicity was recorded using the 2011 Census question, and recoded as ‘White’ or ‘Black, Asian, and Minority Ethnic’ (BAME; any non-White or mixed ethnic background). Marital status was recorded as: ‘married or living as married’, ‘single’, and ‘widowed, divorced, or separated’. Social grade, an indicator of socioeconomic status (SES), was recorded in four categories using the National Readership Survey grades, which is based on details regarding the occupation of a household’s chief income earner [30]: ‘AB’ (Higher and intermediate managerial, administrative, and professional), ‘C1’ (Supervisory, clerical and junior managerial, administrative, or professional), ‘C2’ (skilled manual workers), and ‘DE’ (semi-skilled and unskilled manual workers; and state pensioners, casual and lowest grade workers, and unemployed with state benefits only). ‘Don’t know’ or ‘refused’ responses were coded as missing throughout.

### Analyses

We excluded those with a cancer diagnosis or refusing to answer this question, as well as those with missing data on the demographic and general cancer worry variables. We report the descriptive characteristics of the sample, including prevalence numbers, applying weights to the data to adjust for sampling bias in relation to age, gender, government region, social grade, and working status.

We examined the population distribution of general cancer worry and the two specific worries scales using unweighted data for the univariate and multivariate linear regression models. A sensitivity analysis using weighted data did not change the pattern or significance of the results, so we report the unweighted results. We also conducted subgroup analyses to examine whether the demographic pattern of specific cancer worries differed between those who never worry about their chance of getting cancer versus those who worry at least occasionally, using a dichotomised version of the general cancer worry item. Lastly, we examined the correlations between general cancer worry and the specific worries subscales, and compared mean scores using multiple t-tests with a Bonferroni correction for multiple testing. All analyses were conducted in SPSS version 23, and an overall alpha level of 0.05 to indicate significance.

## Results

In total, 2048 respondents were interviewed; after excluding those with a cancer diagnosis (*n* = 97; 4.7%), refusing to answer this question (*n* = 24; 1.2%), or with missing data on the demographic (*n* = 10; 0.5%) or general cancer worry variables (*n* = 27; 1.3%) 1890 were left for analysis (*N* = 1894 after weighting). Weighted sample characteristics are presented in Table 1.

**Table 1:** Characteristics of the weighted samples

	General cancer worry (*N* = 1894)	Specific cancer worries (*N* = 1782)
Characteristic	*N* (%)^a^	*N* (%)^a^
Age		
Mean, SD	42.8 (15.3)	42.9 (15.3)
Range	18–70	18–70
Gender		
Male	936 (49.4)	884 (49.6)
Female	957 (50.6)	898 (50.4)
Ethnicity		
White	1596 (84.3)	1513 (84.9)
BAME^b^	298 (15.7)	269 (15.1)
Social grade		
AB (highest)	505 (26.7)	486 (27.3)
C1	528 (27.9)	499 (28.0)
C2	425 (22.4)	392 (22.0)
DE (lowest)	436 (23.0)	405 (22.7)
Marital status		
Married or living as married	1194 (63.1)	1122 (63.0)
Single	522 (27.5)	490 (27.5)
Widowed, separated, or divorced	178 (9.4)	170 (9.5)
General cancer worry (frequency)		
Never	701 (37.0)	658 (36.9)
Occasionally	657 (34.7)	618 (34.7)
Sometimes	415 (21.9)	390 (21.9)
Often	85 (4.5)	82 (4.6)
Very often	36 (1.9)	34 (1.9)
Specific cancer worries (12 items)		
Complete data	1782 (94.1)	1782 (100.0)
Missing data	112 (5.9)	–

^a^Unless otherwise stated

^b^*BAME* Black, Asian, and Minority Ethnic

### General cancer worry

Just over a third of respondents (37%) never worried about getting cancer, 35% worried occasionally, 22% sometimes, 5% often, and 2% very often. Univariate and multivariate linear regression analyses showed that general cancer worry was less frequent in those who were older and from non-White ethnic backgrounds, and more frequent in women (Table 2). There were no differences by social grade or marital status.

**Table 2:** Mean scores and demographic correlates of general cancer worry (range: 1–5; unweighted data; *N* = 1890)

Characteristic	General cancer worry	Univariate^$^			Multivariate^$,a^		
	M (SD)	B (SE)	β	Significance	B (SE)	β	Significance
Total sample	1.99 (0.98)						
Age		**−.003 (.001)**	**−.053**	***p*** **< .05**	**−.004 (.002)**	**−.071**	***p*** **< .01**
Gender							
Male	1.87 (0.94)	Ref			Ref		
Female	2.10 (1.02)	**.226 (.045)**	**.115**	***p*** **< .001**	**.214 (.046)**	**.108**	***p*** **< .001**
Ethnicity							
White	2.02 (0.98)	Ref			Ref		
BAME^b^	1.88 (1.00)	**−.141 (.062)**	**−.052**	***p*** **< .05**	**−.164 (.063)**	**−.061**	***p*** **< .01**
Social grade							
AB (highest)	2.00 (0.90)	Ref			Ref		
C1	1.97 (0.95)	−.039 (.050)	−.081	*p* = .43	−.039 (.069)	−.018	*p* = .58
C2	2.00 (0.97)	.001 (.055)	.0003	*p* = .99	−.007 (.073)	−.003	*p* = .92
DE (lowest)	2.02 (1.07)	.032 (.048)	.015	*p* = .51	.027 (.069)	.013	*p* = .69
Marital status							
Married or living as married	2.01 (0.97)	Ref			Ref		
Single	1.97 (1.00)	−.034 (.050)	−.016	*p* = .50	−.074 (.055)	−.034	*p* = .18
Widowed/separated/divorced	1.96 (1.04)	−.044 (.073)	−.014	*p* = .55	−.049 (.078)	−.016	*p* = .53

R^2^ = .021

^$^Values in bold are significant at *p* < .05

^a^Mutually adjusted for age, gender, ethnicity, social grade, and marital status

^b^BAME = Black, Asian, and Minority Ethnic

### Specific cancer worries

A further 117 respondents (6.2%) were excluded because of missing data on the specific cancer worries items (Table 1). If diagnosed with cancer, about two thirds said they would be ‘quite a bit’ or ‘extremely’ worried about the *threat to life* and *emotional upset* that a cancer diagnosis would cause (Table 3). About half would be worried about *surgery*, *radiation treatment*, *chemotherapy*, and the *loss of control over life*. Worries about the social implications of a cancer diagnosis were less frequently anticipated: slightly less than half would be worried about *financial problems* or their *social roles*, while only about a quarter would be worried how cancer would affect their *identity*, *important relationships*, and *sexuality*. Only a fifth would worry about how a diagnosis of cancer would affect their *gender role*.

**Table 3:** Response rates and distributions for the specific cancer worry items (weighted; *N* = 1894)

If you were diagnosed with cancer, would you worry that...	Response rate *N* (%)	Not at all *N* (%)	Slightly *N* (%)	Quite a bit *N* (%)	Extremely *N* (%)
Worries about the emotional and physical consequences					
1.you would require surgery?	1796 (94.8)	349 (19.4)	513 (28.6)	592 (33.0)	342 (19.0)
2.it would upset you emotionally?	1799 (95.0)	190 (10.6)	443 (24.6)	616 (34.2)	550 (30.5)
3.you would require chemotherapy?	1796 (94.8)	292 (16.3)	455 (25.3)	659 (36.7)	390 (21.7)
4.you would require radiation treatment?	1799 (95.0)	337 (18.7)	497 (27.6)	636 (35.4)	329 (18.3)
5.it would make you feel that you don’t have control over your life?	1794 (94.7)	335 (18.7)	551 (30.7)	540 (30.1)	368 (20.5)
6.it would threaten your life?	1799 (95.0)	211 (11.7)	373 (20.7)	638 (35.5)	577 (32.1)
Worries about the social consequences					
7.it would make you feel less of a (wo)man?	1799 (95.0)	1044 (58.1)	416 (23.1)	230 (12.8)	109 (6.1)
8.it would interfere with your sense of sexuality?	1800 (95.0)	868 (48.2)	467 (25.9)	326 (18.1)	139 (7.7)
9.it would threaten your identity?	1795 (94.8)	781 (43.5)	507 (28.2)	359 (20.0)	148 (8.2)
10.it would hurt your relationships with friends and family?	1800 (95.0)	814 (45.2)	482 (26.8)	329 (18.3)	175 (9.7)
11.it would cause financial problems for you?	1797 (94.9)	425 (23.6)	510 (28.4)	531 (29.5)	331 (18.4)
12.it would keep you from fulfilling important roles?	1797 (94.9)	361 (20.1)	548 (30.5)	566 (31.5)	322 (17.9)

#### Worries about the emotional and physical consequences

The sample mean for worries about the emotional and physical consequences of a cancer diagnosis was 2.65/4 (SD 0.80), equating to a score between ‘slightly’ and ‘quite a bit’ (Table 4). In univariate and multivariate analyses, women and those who were younger were more likely to anticipate worrying about the emotional and physical consequences of a cancer diagnosis, while those from social grade DE were less likely to anticipate worry about this compared with social grade AB, but there were no differences by ethnicity, marital status, or social grades C1 and C2.

**Table 4:** Mean scores and demographic correlates of worries about the emotional and physical consequences of a cancer diagnosis (range: 1–4; unweighted; *N* = 1773)

Characteristic	Emotional and physical worries	Univariate^$^			Multivariate^$,a^		
	M (SD)	B (SE)	β	Significance	B (SE)	β	Significance
Total sample	2.65 (0.80)						
Age		**−.007 (.001)**	**−.143**	***p*** **< .001**	**−.007 (.001)**	**−.148**	***p*** **< .001**
Gender							
Male	2.51 (0.76)	Ref			Ref		
Female	2.77 (0.82)	**.256 (.038)**	**.159**	***p*** **< .001**	**.246 (.038)**	**.153**	***p*** **< .001**
Ethnicity							
White	2.65 (0.79)	Ref			Ref		
BAME^b^	2.62 (0.86)	−.030 (.053)	−.013	*p* = .57	−.076 (.053)	−.034	*p* = .15
Social grade							
AB (highest)	2.70 (0.77)	Ref			Ref		
C1	2.69 (0.78)	.052 (.042)	.029	*p* = .22	−.050 (.057)	−.028	*p* = .38
C2	2.68 (0.80)	.034 (.046)	.017	*p* = .47	−.042 (.060)	−.022	*p* = .48
DE (lowest)	2.57 (0.84)	**−.113 (.041)**	**−.066**	***p*** **< .01**	**−.155 (.057)**	**−.090**	***p*** **< .01**
Marital status							
Married or living as married	2.64 (0.79)	Ref			Ref		
Single	2.69 (0.84)	.049 (.042)	.028	*p* = .24	.005 (.046)	.003	*p* = .91
Widowed/separated/divorced	2.60 (0.80)	−.052 (.061)	−.020	*p* = .40	.057 (.065)	.022	*p* = .37

R^2^ = .050

^$^Values in bold are significant at *p* < .05

^a^Mutually adjusted for age, gender, ethnicity, social grade, and marital status

^b^BAME = Black, Asian, and Minority Ethnic

Subgroup analyses showed that anticipated worry about the emotional and physical consequences of a cancer diagnosis was significantly higher in those who worried at least occasionally versus those who never worried about their chance of getting cancer (M = 2.86, SD 0.71 vs M = 2.31, SD 0.84; t(1229) = −14.22, *p* < .001). Worry about the emotional and physical consequences was associated with younger age in both groups, and with being female in the group who worry at least occasionally about getting cancer (all *p* < .01; Additional file 3). There were no associations with ethnicity, social grade, or marital status for either group.

#### Worries about the social consequences

The sample mean for worries about the social consequences of a cancer diagnosis was 2.04/4 (SD 0.75), equating to feeling slightly worried (Table 5). Univariate and multivariate analyses showed that women, those who were younger, and those from BAME groups anticipated greater worry about the social consequences. Those who were single would be more worried about the social consequences of a cancer diagnosis than those who were married in unadjusted analyses, but this relationship was attenuated by adjusting for other demographic differences. There was no association with social grade.

**Table 5:** Mean scores and demographic correlates of worries about the social consequences of a cancer diagnosis (range: 1–4; unweighted; N = 1773)

Characteristic	Social worries	Univariate^$^			Multivariate^$,a^		
	M (SD)	B (SE)	β	Significance	B (SE)	β	Significance
Total sample	2.04 (0.75)						
Age		**−.011 (.001)**	**−.239**	***p*** **< .001**	**−.011 (.001)**	**−.226**	***p*** **< .001**
Gender							
Male	1.95 (0.72)	Ref			Ref		
Female	2.12 (0.77)	**.171 (.035)**	**.114**	***p*** **< .001**	**.160 (.035)**	**.107**	***p*** **< .001**
Ethnicity							
White	2.01 (0.73)	Ref			Ref		
BAME^b^	2.24 (0.83)	**.226 (.049)**	**.108**	***p*** **< .001**	**.153 (.049)**	**.073**	***p*** **< .01**
Social grade							
AB (highest)	1.99 (0.72)	Ref			Ref		
C1	2.05 (0.74)	.009 (.039)	.006	*p* = .82	−.010 (.052)	−.006	*p* = .84
C2	2.11 (0.74)	.083 (.043)	.046	*p* = .05	.086 (.055)	.047	*p* = .12
DE (lowest)	2.02 (0.78)	−.034 (.038)	−.021	*p* = .38	−.012 (.052)	−.008	*p* = .82
Marital status							
Married or living as married	2.02 (0.73)	Ref			Ref		
Single	2.13 (0.78)	**.128 (.039)**	**.078**	***p*** **< .01**	.006 (.042)	.003	*p* = .90
Widowed/separated/divorced	1.95 (0.75)	−.109 (.057)	−.045	*p* = .06	.047 (.059)	.019	*p* = .43

R^2^ = .076

^$^Values in bold are significant at *p* < .05

^a^Mutually adjusted for age, gender, ethnicity, social grade, and marital status

^b^BAME = Black, Asian, and Minority Ethnic

Subgroup analyses showed that anticipated worry about the social consequences of a cancer diagnosis was significantly higher in those who worried at least occasionally compared with those who never worried about their chance of getting cancer (M = 2.20, SD 0.73 vs M = 1.78, SD 0.71; t(1771) = −11.81, *p* < .001). In both groups, being worried about the social consequences of a cancer diagnosis was associated with younger age (Additional file 3). For those who worried at least occasionally about getting cancer, worry about the social consequences was also associated with being female and being from an ethnic minority background. As per the main analyses, there were no associations with social grade or marital status.

### The relationships between general cancer worry and the specific cancer worries

Anticipated worry about the emotional and physical consequences of a cancer diagnosis (M = 2.65/4, SD = 0.80) was significantly higher than worry about the social consequences of a cancer diagnosis (M = 2.04/4, SD = 0.75; t(1772) = 41.2, *p* < .001), or general cancer worry (M = 2.00/5, SD = 0.98; t(1772) = 26.6, *p* < .001). There was no difference between the latter two (t(1772) = 1.81, *p* = .07). The correlations between general cancer worry and the two specific cancer worries sub-scales were moderate: *r* = .349 (*p* < .001; emotional and physical consequences) and *r* = .305 (*p* < .001; social consequences). However, the two sub-scales of specific cancer worries were strongly correlated (Pearson’s *r* = .683, *p* < .001).

## Discussion

This is the first study to examine the prevalence and demographic distribution of specific worries about cancer in the general population, and to compare this with general cancer worry. Nearly two thirds of the English adult population without a cancer diagnosis worry at least occasionally about getting cancer, and a fifth to two thirds anticipated worrying about specific aspects of the cancer experience if they were diagnosed. The population distributions for these worries partly overlap, but there may be important implications for future research where they diverge, as described below.

The prevalence and population distribution of general cancer worry in our study was similar to other population-based studies in the UK and US [25, 27, 31], with higher general cancer worry in women and those who are younger [25–27, 32]. The gender difference in cancer worry may be partly explained by women being more anxious in general [33], although previous studies found only moderate correlations between cancer worry and general anxiety [16, 27]. Little is known about the negative association of cancer worry with age. Possible explanations include greater familiarity with cancer and cancer outcomes in others (including positive ones), or less fear of cancer outcomes such as disability and death due to advanced age. Some studies have shown that older adults are indeed less fearful of death [34], and 40% of older adults in England believe that cancer is a good way to die [35]. Unlike previous studies [16, 27], we found that general cancer worry was lower among those from BAME backgrounds. However, levels of worry vary between ethnic groups [26, 32, 36], and may thus depend on the representation of specific ethnic groups in the sample. We could not explore this further due to small numbers in the ethnic groups.

In terms of the specific cancer worries, more people would be worried about the emotional and physical consequences of a cancer diagnosis than about the social consequences, although the latter were still reported by a fifth to half the population. This is the first study to examine specific cancer worries in the general population, although a study in breast cancer survivors and a qualitative review of cancer worries in cancer screening also found higher anticipation of treatment and death worries than worries about cancer’s social implications [20, 23]. Many people believe that ‘a diagnosis of cancer is a death sentence’ and that ‘cancer treatment is worse than the cancer itself’, which may explain this finding [37, 38]. We also noted the high prevalence of worry about financial consequences (76% would worry at least slightly), despite healthcare in England being provided free at the point of delivery via the National Health Service. Cancer patients often incur many indirect costs, however, such as for transportation, childcare, and lost wages [39], and the high prevalence of anticipated financial worry may reflect this.

This study has the following implications for future research. First, the population distribution of the specific worries about cancer was similar to that of general cancer worry, except for ethnicity. While we found no ethnic differences in worries about emotional and physical consequences, those from BAME backgrounds worried *more* about the social consequences of a cancer diagnosis. This could reflect cancer being taboo or stigmatised in some ethnic minority populations [40–42], and may help explain their lower uptake of cancer screening [43–45], and tendency to delay help-seeking for symptoms [46]. Future research into the behavioural effects of cancer worry in ethnic minority populations may benefit from distinguishing general cancer worry from specific worries about the social consequences of a cancer diagnosis.

Secondly, previous studies suggest that general cancer worry sometimes motivates help-seeking and attendance at cancer screening [10], but specific cancer worries may act as a deterrent [11, 23]. Although we did not assess the behavioural associations of the cancer worries included in this study, we did show that general cancer worry and specific cancer worries are only moderately correlated, and it may be important to distinguish them when assessing their behavioural effects. For example, behavioural theories such as the EPPM [14] would predict that the behavioural responses for general cancer worry and specific worries may be different: general cancer worry may motivate cancer screening attendance to obtain reassurance to deal with the threat of cancer, while fears about cancer treatment may motivate avoidance of screening to avoid a diagnosis, thereby dealing with the threat of cancer treatment. Given that 50% of older adults in the UK believe that most cancer treatment is worse than the cancer itself [38], distinguishing between the specific origins of cancer worry may help determine when cancer worry acts as a motivator and when it acts as a deterrent to cancer preventive behaviours. Assessing the behavioural effects of specific cancer worries was outside the scope of this study, however, and further research into these behavioural effects is needed.

Our study had some limitations. The specific worry items used were from a scale that had been previously developed and validated with breast cancer patients [20]. Although we used cognitive interviewing and an online pilot study to ensure items were clear and relevant to people without a cancer diagnosis, more work is needed to fully validate the scale for use in the general population. This study is only a first step in the exploration of specific cancer worries in the general population, and future research into specific cancer worries and their behavioural effects would benefit from further validation of this scale. Furthermore, a substantial proportion of respondents (7%) had missing data on the specific cancer worry items, which could have influenced estimates of their prevalence and population distribution. However, if those who worry are more likely to avoid answering these questions, then our prevalence estimates may be an underestimate. The cross-sectional nature of this study prevents any conclusions about temporal or causal relationships (such as whether cancer worry decreases with age), and longitudinal studies would be needed to examine how cancer worries evolve over time. Cancer worries may be affected by family history of cancer, but we were unable to measure this in the current study and future studies may want to explore this relationship. Finally, we aimed to examine whether there were differences in the prevalence and sociodemographic distribution of twelve specific worries about cancer. We did not specify a specific type of cancer, although the importance of these worries may vary by cancer type, for example, depending on the treatment options and survivability of a particular cancer type. Although this was outside of the scope of the current study, future studies could explore whether the prevalence of specific cancer worries varies by cancer type.

## Conclusion

In conclusion, many aspects of cancer may evoke worry in the general population, including general worry about getting cancer and anticipated worries about the emotional, physical, and social consequences of a cancer diagnosis. This study can serve as a starting point to examine the behavioural effects of different cancer worries, and may ultimately help allay undue worries in those who are deterred by them.

## Additional files

Additional file 1: Adaptation of items from the Concerns About Recurrence Scale for use in a general population sample. (DOC 86 kb)

Additional file 2: Specific cancer worry items, factor loadings, and Cronbach’s alpha for each sub scale. (DOC 36 kb)

Additional file 3: Multivariate regression analyses by general cancer worry (‘never’ vs ‘at least occasionally’). (DOC 57 kb)

## Abbreviations

ABACUS: Attitudes, Behaviour and Cancer UK Survey
BAME: Black, Asian, and Minority Ethnic
CARS: Concerns About Recurrence Scale
EPPM: Extended Parallel Process Model
SES: Socioeconomic status

## Footnotes

1 In population research, the terms ‘cancer fear’ and ‘cancer worry’ are often used interchangeably. In clinical psychology, ‘fear’ refers to the emotional response to an immediate and observable threat and ‘worry’ refers to a more cognitive process where the threat is often anticipatory in nature [Rachman, S. Anxiety, 2nd ed. Hove: Psychology Press; 2004]. Consistent with these definitions, we will use the term ‘cancer worry’ to describe the negative emotional reaction to the threat of cancer in an asymptomatic population, except when referring to previous studies, where we will use the term that is used by the authors.
