# Supplementary material for: What is it about a cancer diagnosis that would worry people? A population-based survey of adults in England
Source: BMC Cancer. 2018 Jan 24;18:86. doi: 10.1186/s12885-017-3963-4 (PMC5781324; doi:10.1186/s12885-017-3963-4)
Supplement: Supplementary file 1 — Adaptation of items from the Concerns About Recurrence Scale for use in a general population sample. (DOC 86 kb) [file 12885_2017_3963_MOESM1_ESM.doc]

**What is it about cancer that worries people? A population-based survey of adults in England**

By Philippa J Murphy, Laura A.V Marlow, Jo Waller, and Charlotte Vrinten

**Online Supplement 1. Adaptation of items from the Concerns About Recurrence Scale for use in a general population sample**

This online supplement to “What is it about cancer that worries people? A population-based survey of adults in England” by Philippa J. Murphy, Laura A.V Marlow, Jo Waller and Charlotte Vrinten describes how Vickberg’s Concerns About Recurrence Scale (Vickberg, 2003) was adapted and shortened to measure specific worries about cancer in a population-based sample without a previous diagnosis of cancer.

**Vickberg’s Concerns About Recurrence Scale**

The Concerns About Recurrence Scale (CARS) was developed by Suzanne Vickberg to assess worries about breast cancer recurrence in female breast cancer survivors (Vickberg, 2003). It consists of 30 items; four of which assess overall worry about cancer recurrence, and 26 items assess the nature of women’s worries about cancer recurrence. These 26 items are divided into four sub-scales, and are measured on a 5-point response scale (not at all, a little, moderately, a lot, extremely).

- *Health worries* (11 items), e.g. “I worry that a recurrence of breast cancer would require further surgery”
- *Womanhood worries* (7 items), e.g. “I worry that a recurrence of breast cancer would interfere with my sense of sexuality”
- *Role worries* (6 items), e.g. “I worry that a recurrence of breast cancer would keep me from fulfilling important roles (in my home or at my job)”
- *Death worries* (2 items), e.g. “I worry that a recurrence of breast cancer would threaten my life”

During the development of this scale, face validity of the items was assessed in a pilot study with 16 breast cancer survivors, two nurses, two surgeons, and one social worker working with breast cancer patients. The CARS was subsequently validated in a sample of 169 breast cancer survivors using exploratory factor analysis. Internal reliability was high, with Cronbach’s alphas for the four sub-scales ranging from .89 to .94. Convergent validity was demonstrated with the Intrusive Thoughts and Avoidance subscales of the Impact of Events scale, as well as the Distress and Well-Being scales of the Mental Health Inventory. Test-retest reliability was not assessed, nor was a confirmatory factor analysis done.

**Adaptation of the CARS**

To our knowledge, this is the first time that items from the CARS have been adapted for use in the general population. Other multidimensional scales of fear of cancer recurrence exist, for example, the Fear of Progression Questionnaire (Herschbach *et al*, 2005) and the Fear of Cancer Recurrence Inventory (Simard and Savard, 2009), but we chose to adapt the CARS for two reasons. First, the CARS items needed less adapting because they were already phrased as anticipated worries about a future event, rather than worries about current illness (such as in Herschbach *et al*, 2005). Secondly, the items seemed to best represent the various concerns about cancer that people in the general population have, as identified by a recent qualitative review (Vrinten *et al*, 2016), although other fear of recurrence scales also show some thematic overlap (e.g. Herschbach *et al*, 2005).

**Online pilot study methods**

For our study, we only adapted the 26 CARS items about specific cancer worries (and not the 4 items about general worry of cancer recurrence). We first adjusted the wording of these items to make them suitable to assess worries about cancer in general (rather than breast cancer) in a population-based sample of respondents consisting of both genders and without a previous cancer diagnosis. For example, the item “I worry that a recurrence of breast cancer would make me feel I am less of a woman” (Vickberg, 2003) was changed to “Do you worry that if you were diagnosed with cancer, it would make you feel less of a man or a woman?” At this stage, the following two items were dropped from the scale because they were deemed too specific to having a previous diagnosis of (breast) cancer: “I worry that a recurrence of breast cancer would be more serious than the first” and “I worry that a recurrence of breast cancer would mean losing my breast”. In addition, the 5-point response scale was changed to a 4-point response scale (1: not at all, 2: slightly, 3: quite a bit, 4: extremely) to better reflect natural language and for ease of interpretation.

We then tested the 24 remaining items in an online survey of population-based adults aged 18-70 years in England to reduce the number of items for the face-to-face ABACUS survey due to space restrictions for this survey. For this online pilot survey, we recruited a sample via the online panel of market research agency Survey Sampling International, using quota sampling based on age, gender, and highest level of educational qualification to approach the sample characteristics of the face-to-face national ABACUS survey. Survey Sampling International offered participants who completed the survey a reward in the form of cash or gift vouchers. Ethical approval for the ABACUS survey including the online pilot survey was obtained from the UCL Research Ethics Committee prior to recruitment (registration number: 5771/002), and participants were asked to consent to participate at the start of the online survey. Those with a previous diagnosis of cancer were excluded from participation.

In addition to demographic data including gender, age, educational qualification, and ethnicity, respondents were asked about general worry about cancer (“How often do you worry about your chance of getting cancer yourself?”) with response options on a 5-point scale (“never”, “occasionally”, “sometimes”, “often”, “very often”). Only those who worried at least occasionally about their chance of getting cancer were asked the 24 adapted CARS items.

We used SPSS version 23 to perform an exploratory factor analysis of these 24 items using principal component analysis and an oblique rotation because the resulting factors were expected to correlate (Vickberg, 2003). We then examined the factor structure and factor loadings for each item, and the item-total and inter-item correlations for each sub-scale to assess which items might be statistically redundant. Two authors (CV and JW) collated this information with the factor structure of the original CARS (Vickberg, 2003), and the different aspects of cancer fear as found in the general population (Vrinten *et al*, 2016), to decide which items to retain for the face-to-face ABACUS survey.

**Results**

In total, 526 participants started the online survey. Of these, 73 participants were excluded before reaching the main part of the survey (16 did not consent to participate, 48 were diagnosed with cancer, 4 did not meet the age eligibility criteria, and 5 dropped out at this point). A further 22 participants dropped out during the survey. Quality checks such as for ‘speeding’ (defined as completing the survey in less than half of the median time-to-complete) resulted in another 39 participants being excluded. Of the remaining 392 participants, 60 (15.3%) never worried about their chances of getting cancer and were thus excluded from the adapted CARS items, leaving a sample of 332 for the exploratory factor analysis.

The KMO statistic was .941 and Bartlett’s test of sphericity was significant, indicating that the data were suitable for factor analysis. Inspection of the Eigenvalues and scree plot suggested a 3-factor solution, which together explained 62.9% of the variance. The Eigenvalue for Factor 1 was 11.12 and this factor explained 46.3% of the variance. The Eigenvalue for Factor 2 was 2.76 and this explained 11.5% of the variance, and the Eigenvalue for Factor 3 was 1.22, which explained 5.1% of the variance. The factor structure for this population-based sample showed a large overlap with the factor structure in breast cancer survivors using the original CARS scale (Vickberg, 2003), except for the Death worries items. These did not emerge as a separate factor in our analysis, possibly because there were only two items in this subscale. Factor loadings for each item, and Cronbach’s alpha and item-total correlations for the items in each sub-scale, are presented in Table 1.

Table 1. Factor loadings, and Cronbach’s alpha and item-total correlations for each subscale in a population-based sample (n=332), and original CARS factor structure.

| **Item**  **Do you worry that if you were diagnosed with cancer** | **Factor 1*** | **Factor 2*** | **Factor 3*** | **Item-total correlation** | **Original CARS subscale** |
| --- | --- | --- | --- | --- | --- |
| *Health worries sub-scale* |  |  |  |  |  |
| It would threaten your life? | **.899** | -.114 | -.020 | .785 | Death worries |
| It would threaten your physical health? | **.857** | -.159 | -.178 | .749 | Health worries |
| You would require chemotherapy? | **.811** | .103 | .218 | .773 | Health worries |
| It would cause pain and suffering? | **.806** | -.028 | .056 | .719 | Health worries |
| It would cause you to die? | **.804** | -.091 | .055 | .668 | Death worries |
| It would make you feel you don’t have control over your life? | **.694** | .187 | -.096 | .768 | Health worries |
| You would require radiation treatment? | **.693** | .242 | .298 | .712 | Health worries |
| It would interfere with your ability to plan for the future? | **.678** | .105 | -.275 | .738 | Health worries |
| It would interfere with your physical ability to carry out daily activities? | **.602** | .160 | -.374 | .697 | Role worries |
| It would keep you from doing the things you had planned to? | **.547** | .145 | -.344 | .640 | Health worries |
| It would upset you emotionally? | **.541** | .272 | .075 | .622 | Health worries |
| You would require surgery? | **.495** | .153 | .482 | .424 | Health worries |
| *Role worries sub-scale* |  |  |  |  |  |
| It would make you feel less of a man or a woman? | .043 | **.837** | .085 | .788 | Womanhood worries |
| It would make you feel less feminine or masculine? | -.052 | **.810** | -.020 | .713 | Womanhood worries |
| It would interfere with your sense of sexuality? | .021 | **.789** | .060 | .746 | Womanhood worries |
| It would threaten your identity (how you see yourself)? | .158 | **.771** | -.001 | .800 | Womanhood worries |
| It would threaten your spirituality or faith? | -.242 | **.756** | .092 | .504 | Womanhood worries |
| It would make you feel badly about how your body looks or feels? | .175 | **.730** | -.003 | .768 | Womanhood worries |
| It would damage your romantic relationships? | -.060 | **.725** | -.182 | .657 | Womanhood worries |
| It would hurt your relationship with friends and family? | .073 | **.667** | -.106 | .662 | Role worries |
| It would harm your self-confidence? | .279 | **.664** | .003 | .768 | Role worries |
| It would cause financial problems for you? | .312 | **.392** | -.199 | .540 | Role worries |
| *Responsibility worries sub-scale* | | | | | |
| It would keep you from fulfilling your responsibilities (in your home or at your job)? | .217 | .365 | **-.557** | .732 | Role worries |
| It would keep you from fulfilling important roles (in your home or at your job)? | .384 | .399 | **-.424** | .732 | Role worries |
| Cronbach’s alpha | .93 | .92 | .85 |  |  |

* Numbers in bold represent the highest factor loadings.

We then examined the inter-item correlations for the items in each subscale to assess possible redundancy (results not shown), and collated these results with the various aspects of cancer fear as found in our systematic review and meta-synthesis of cancer fears in the general population (Vrinten *et al*, 2016). We retained those items with the highest item-total correlation, lowest inter-item correlation, and which were deemed to represent an important aspect of worry about cancer. For example, the ‘worry about cancer surgery’ item was retained despite a low item-total correlation, because this was found to be an important and distinct cancer-related fear in the meta-synthesis (Vrinten *et al*, 2016). Twelve items were retained for the face-to-face ABACUS survey (see Table 1 in manuscript).

**Abbreviations**

ABACUS Attitudes, Behaviour, and Cancer UK Survey

CARS Concerns About Recurrence Scale

**References**

Herschbach P, Berg P, Dankert A, Duran G, Engst-Hastreiter U, Waadt S, et al.

Fear of progression in chronic diseases: psychometric properties of the Fear of Progression Questionnaire. *J Psychosom Res* **2005**; 58:505-511.

Simard S, Savard J. Fear of cancer recurrende inventory: development and initial

validation of a multidimensional measure of fear of cancer recurrence. *Support Care Cencer* **2009**;17:241-251.

Vickberg SMJ. The concerns about recurrence scale (CARS): A systematic

measure of women’s fears about the possibility of breast cancer recurrence. *Ann Behav Med* **2003**;17:241-51.

Vrinten C, McGregor LM, Heinrich M, von Wagner C, Waller J, Wardle J, et al.

What do people fear about cancer? A systematic review and meta-synthesis of cancer fears in the general population. *Psychooncology* **2016**; Epub 19 September 2016.
