# Supplementary material for: What is it about a cancer diagnosis that would worry people? A population-based survey of adults in England
Source: BMC Cancer. 2018 Jan 24;18:86. doi: 10.1186/s12885-017-3963-4 (PMC5781324; doi:10.1186/s12885-017-3963-4)
Supplement: Supplementary file 2 — Specific cancer worry items, factor loadings, and Cronbach’s alpha for each sub scale. (DOC 36 kb) [file 12885_2017_3963_MOESM2_ESM.doc]

**What is it about cancer that worries people? A population-based survey of adults in England**

By Philippa J Murphy, Laura A.V Marlow, Jo Waller, and Charlotte Vrinten

**Online Supplement 2.**

Specific cancer worry items, factor loadings, and Cronbach’s alpha for each sub scale

| **If you were diagnosed with cancer, would you worry that…** | **Factor 1^*^** | **Factor 2^*^** |
| --- | --- | --- |
| *Worries about the emotional and physical consequences (factor 1)* |  |  |
| 1 …you would require surgery? | **.787** | .001 |
| 2 …it would upset you emotionally? | **.728** | .067 |
| 3 …you would require chemotherapy? | **.906** | -.102 |
| 4 …you would require radiation treatment? | **.860** | -.054 |
| 5 …it would make you feel that you don’t have control over your life? | **.541** | .320 |
| 6 …it would threaten your life? | **.761** | .047 |
| *Worries about the social consequences (factor 2)* |  |  |
| 7 …it would make you feel less of a man or a woman? | -.104 | **.887** |
| 8 …it would interfere with your sense of sexuality? | -.019 | **.785** |
| 9 …it would threaten your identity (how you see yourself)? | .096 | **.738** |
| 10 …it would hurt your relationships with your friends and family? | -.023 | **.798** |
| 11 …it would cause financial problems for you? | .282 | **.473** |
| 12 …it would keep you from fulfilling important roles (in your home or at your job)? | .375 | **.423** |
| **Cronbach’s alpha for each sub scale** | **0.88** | **0.85** |

*Bold numbers indicate the strongest factor loading and the sub-scale on which this item was retained.
