# Supplementary material for: What is it about a cancer diagnosis that would worry people? A population-based survey of adults in England
Source: BMC Cancer. 2018 Jan 24;18:86. doi: 10.1186/s12885-017-3963-4 (PMC5781324; doi:10.1186/s12885-017-3963-4)
Supplement: Supplementary file 3 — Multivariate regression analyses by general cancer worry (‘never’ vs ‘at least occasionally’). (DOC 57 kb) [file 12885_2017_3963_MOESM3_ESM.doc]

**What is it about cancer that worries people? A population-based survey of adults in England**

By Philippa J Murphy, Laura A.V Marlow, Jo Waller, and Charlotte Vrinten

**Online Supplement 3. Multivariate regression analyses by general cancer worry (‘never’ vs ‘at least occasionally’)**

Table A. Mean scores of worries about the emotional and physical consequences of a cancer diagnosis (range: 1-4), and multivariate linear regression analyses for sociodemographic correlates (unweighted), for the whole sample (N=1,773) and split by worrying ‘never’ (N=669) vs ’at least occasionally’ (N=1,104) about getting cancer

| **Characteristic** | Total sample | | | | ‘Never’ worry | | | | Worry ‘at least occasionally’ | | | |
| --- | --- | --- | --- | --- | --- | --- | --- | --- | --- | --- | --- | --- |
|  | **M (SD)** | **B (SE)** | **β** | **Sign.** | **M (SD)** | **B (SE)** | **β** | **Sign.** | **M (SD)** | **B (SE)** | **β** | **Sign.** |
| Total sample | 2.65 (0.80) |  |  |  | 2.31 (0.84) |  |  |  | 2.86 (0.71) |  |  |  |
| Age |  | **-.007 (.001)** | **-.148** | **p<.001** |  | **-.006 (.002)** | **-.120** | **p=.008** |  | **-.007 (.002)** | **-.157** | **p<.001** |
| Gender  Male  Female | 2.51 (0.76)  2.77 (0.82) | Ref  **.246 (.038)** | **.153** | **p<.001** | 2.26 (0.79)  2.37 (0.89) | Ref  .115 (.065) | .069 | p=.08 | 2.71 (0.58)  2.97 (0.70) | Ref  **.238 (.042)** | **.166** | **p<.001** |
| Ethnicity  White  BAME | 2.65 (0.79)  2.62 (0.86) | Ref  -.076 (.053) | -.034 | p=.15 | 2.31 (0.83)  2.29 (0.89) | Ref  -.088 (.085) | -.041 | p=.30 | 2.85 (0.71)  2.92 (0.71) | Ref  .033 (.063) | .016 | p=.60 |
| Social grade  AB (highest)  C1  C2  DE (lowest) | 2.70 (0.77)  2.69 (0.78)  2.68 (0.80)  2.57 (0.84) | Ref  -.050 (.057)  -.042 (.060)  **-.155 (.057)** | -.028  -.022  **-.090** | p=.38  p=.48  **p<.01** | 2.32 (0.82)  2.44 (0.78)  2.31 (0.87)  2.18 (0.86) | Ref  .075 (.103)  -.061 (.108)  -.169 (.102) | .041  -.030  -.096 | p=.47  p=.57  p=.10 | 2.88 (0.68)  2.84 (0.74)  2.90 (0.67)  2.83 (0.72) | Ref  -.080 (.062)  .017 (.065)  -.088 (.062) | -.051  .010  -.058 | p=.20  p=.80  p=.16 |
| Marital status  Married/living as  Single  Wid./sep./div. | 2.64 (0.79)  2.69 (0.84)  2.60 (0.80) | Ref  .005 (.046)  .057 (.065) | .003  .022 | p=.91  p=.37 | 2.30 (0.82)  2.35 (0.90)  2.24 (0.77) | Ref  .019 (.078)  .046 (.106) | .010  .018 | p=.81  p=.67 | 2.83 (0.70)  2.92 (0.71)  2.88 (0.70) | Ref  .037 (.052)  .120 (.074) | .023  .051 | p=.48  p=.10 |

^$^ Values in bold are significant at p<.05

Abbreviations: BAME = Black, Asian, and Minority Ethnic; Div.= divorced; SD = standard deviation; SE = standard error; Sep. = separated; Sign. = significance; Wid. = widowed

Table B. Mean scores of worries about the social consequences of a cancer diagnosis (range: 1-4), and multivariate linear regression analyses for sociodemographic correlates (unweighted), for the whole sample (N=1,773) and split by worrying ‘never’ (N=669) vs ’at least occasionally’ (N=1,104) about getting cancer

| **Characteristic** |  | Total sample | | |  | ‘Never’ worry | | |  | Worry ‘at least occasionally’ | | |
| --- | --- | --- | --- | --- | --- | --- | --- | --- | --- | --- | --- | --- |
|  | **M (SD)** | **B (SE)** | **β** | **Sign.** | **M (SD)** | **B (SE)** | **β** | **Sign.** | **M (SD)** | **B (SE)** | **β** | **Sign.** |
| Total sample | 2.04 (0.75) |  |  |  | 1.78 (0.71) |  |  |  | 2.20 (0.73) |  |  |  |
| Age |  | **-.011 (.001)** | **-.226** | **p<.001** |  | **-.009 (.002)** | **-.200** | **p<.001** |  | **-.011 (.002)** | **-.233** | **p<.001** |
| Gender  Male  Female | 1.95 (0.72)  2.12 (0.77) | Ref  **.160 (.035)** | **.107** | **p<.001** | 1.76 (0.68)  1.81 (0.75) | Ref  .062 (.054) | .044 | p=.25 | 2.10 (0.71)  2.27 (0.73) | **Ref**  **.152 (.043)** | **.103** | **p<.001** |
| Ethnicity  White  BAME^§^ | 2.01 (0.73)  2.24 (0.83) | Ref  **.153 (.049)** | **.073** | **p<.01** | 1.75 (0.68)  1.95 (0.81) | Ref  .129 (.071) | .071 | p=.07 | 2.16 (0.71)  2.48 (0.78) | **Ref**  **.258 (.063)** | **.119** | **p<.001** |
| Social grade  AB (highest)  C1  C2  DE (lowest) | 1.99 (0.72)  2.05 (0.74)  2.11 (0.74)  2.02 (0.78) | Ref  -.010 (.052)  .086 (.055)  -.012 (.052) | -.006  .047  -.008 | p=.84  p=.12  p=.82 | 1.70 (0.65)  1.81 (0.68)  1.87 (0.75)  1.74 (0.73) | Ref  .025 (.086)  .116 (.090)  -.004 (.085) | .016  .067  -.003 | p=.77  p=.20  p=.97 | 2.13 (0.71)  1.20 (0.74)  2.25 (0.69)  2.21 (0.76) | Ref  .005 (.063)  .106 (.066)  .025 (.063) | .003  .060  .016 | p=.93  p=.11  p=.69 |
| Marital status  Married/living as  Single  Wid./sep./div. | 2.02 (0.73)  2.13 (0.78)  1.95 (0.75) | Ref  .006 (.042)  .047 (.059) | .003  .019 | p=.90  p=.43 | 1.76 (0.70)  1.89 (0.75)  1.63 (0.59) | Ref  .033 (.065)  -.008 (.089) | .022  -.004 | p=.61  p=.93 | 2.16 (0.70)  2.30 (0.76)  2.18 (0.78) | Ref  .019 (.052)  .125 (.075) | .012  .052 | p=.72  p=.10 |

^$^ Values in bold are significant at p<.05

^§^BAME = Black, Asian, and Minority Ethnic
